# Accumulation of *Tetrahymena pyriformis* on Interfaces

**DOI:** 10.3390/mi12111339

**Published:** 2021-10-30

**Authors:** Kohei Okuyama, Yukinori Nishigami, Takuya Ohmura, Masatoshi Ichikawa

**Affiliations:** 1 Department of Physics, Kyoto University, Kyoto 606-8502, Japan; okuyama@kyoryu.scphys.kyoto-u.ac.jp; 2 Research Institute for Electronic Science, Hokkaido University, Sapporo 001-0020, Japan; nishigami@es.hokudai.ac.jp; 3 Biozentrum, University of Basel, 4056 Basel, Switzerland; takuya.ohmura@unibas.ch

**Keywords:** swimming microorganism, ciliates, cell suspension, cell accumulation, diffusion equation

## Abstract

The behavior of ciliates has been studied for many years through environmental biology and the ethology of microorganisms, and recent hydrodynamic studies of microswimmers have greatly advanced our understanding of the behavioral dynamics at the single-cell level. However, the association between single-cell dynamics captured by microscopic observation and pattern dynamics obtained by macroscopic observation is not always obvious. Hence, to bridge the gap between the two, there is a need for experimental results on swarming dynamics at the mesoscopic scale. In this study, we investigated the spatial population dynamics of the ciliate, *Tetrahymena pyriformis*, based on quantitative data analysis. We combined the image processing of 3D micrographs and machine learning to obtain the positional data of individual cells of *T. pyriformis* and examined their statistical properties based on spatio-temporal data. According to the 3D spatial distribution of cells and their temporal evolution, cells accumulated both on the solid wall at the bottom surface and underneath the air–liquid interface at the top. Furthermore, we quantitatively clarified the difference in accumulation levels between the bulk and the interface by creating a simple behavioral model that incorporated quantitative accumulation coefficients in its solution. The accumulation coefficients can be compared under different conditions and between different species.

## 1. Introduction

In recent years, studies have shown that the diversity of eukaryotes is supported by unicellular eukaryotes (protists) [1,2,3]. Protists have diverse genetic backgrounds and play different roles in the environment [4,5]. In other words, the behavior of these microorganisms impact our lives through the food chain of the ecosystem. Swimming unicellular eukaryotes belonging to the ciliate group are the ubiquitous organisms found in fresh, brackish, and coastal waters [6,7]. In the environment, they consume chemicals and smaller bacteria such as protists, and are preyed upon by larger organisms, contributing significantly to the energy cycle. Moreover, the distribution of ciliates is an essential factor in the environment, and the behavior of individual cells realizes such distribution. Therefore, to understand the environment, it is essential to comprehend the behavioral aspects of ciliates.

*Tetrahymena*, a ciliate, has long been studied in various fields (i.e., cell division, the function of mitochondria, glyconeogenesis, the structure of cilia, and ciliated genetics) as an organism that is easy to handle experimentally [8,9]. *Tetrahymena* changes its behavior depending on the external environment. *Tetrahymena* moves toward preferred chemicals such as amino acids and proteins [10,11,12]. In addition, it has recently been reported that *Tetrahymena* changes its behavior according to extracellular space conditions other than chemicals. For example, *Tetrahymena* memorizes the space by confining itself to a small cylindrical area [13,14], assembles at the oxygen-rich air–liquid interface [15,16] or at the food-rich solid–liquid interface [17], and swims against the external flow [18].

In addition to biological experiments, the squirmer model based on fluid dynamics has been used to study the mechanisms of the swimming behaviors of these organisms [19,20,21,22,23,24,25,26,27,28,29]. In the squirmer model, microswimmers are classified into three groups (pusher, puller, and neutral swimmer) according to the distribution of their flow fields around their bodies [30]. Pushers and pullers move closer to the interface [24,25,26,27,28,29], whereas those of the neutral swimmer group, to which *Tetrahymena* belongs, move away from the wall. However, as mentioned above, it has been reported that *Tetrahymena* stays at the air–liquid [15,16] as well as the solid–liquid interfaces [17]. This difference between *Tetrahymena* and a neutral swimmer has been verified experimentally and numerically [16,17]. The accumulation in the air–liquid and solid–liquid interfaces can be reproduced numerically by considering the cell shape and the termination of ciliary beating. The contact between the cell and a wall suppresses ciliary beating and induces the asymmetry of the thrust force around the cell. Currently, the research on the interaction between swimming microorganisms and the interface has been advanced, where the understanding of swimmers at the single-cell level and the habitat distribution of the microorganisms preferring the interface have been compared. However, it is non-trivial to link their individual properties to their macroscopic habitat directly because there are nonlinear phenomena induced by many body interactions composed of several up to sub-million cells, such as mesoscale turbulence in active matter [31,32], collective accumulation [27,33,34], and bioconvection [35,36,37] between the single-cell scale and the macroscopic scale. To understand the complete behavior, from the micro to the macro-scale, it is necessary to elucidate the nonlinear dynamics at the mesoscopic scale, which mediates between macroscopic and unicellular phenomena.

In this article, we tried to elucidate the dynamics of the distribution of Tetrahymena at the mesoscopic scale. The population distribution of *T. pyriformis* in water was captured by 3D imaging, and by using machine learning-based image processing, the spatio-temporal population distribution was successfully quantified. We confirmed the phenomenon of retention at both air–liquid and solid–liquid interfaces using the statistical data of the population. Furthermore, a model that quantitatively describes the accumulation coefficients and compares them with those of different species and under different conditions is suggested.

## 2. Materials and Methods

### 2.1. Preparation of Cells

*T. pyriformis* was cultured in a growth medium of 0.7% (*w*/*v*) Bacto Proteose Peptone (212010, Thermo Fisher, Waltham, MA, USA), 0.2% (*w*/*v*) Paticase (GS1-128, Kyokuto, Japan), and 0.1% (*w*/*v*) Bacto Yeast Extract (212750, Thermo Fisher, Waltham, MA, USA) at room temperature (20–25 °C) without shaking. Before observation, the medium with the cells in the mid-log phase was centrifuged (700× *g* for 6 min), and then the supernatant was removed and preserved for dilution. The cell-containing fraction was divided into six portions, and they were remixed with the preserved supernatant in order to make concentration variations of the observation solutions as ×1, ×2, and ×4. The solution was equilibrated for more than 30 min prior to the observation. The concentration of the cells in each sample was measured using a microscope, as described later in Section 2.3.

### 2.2. Observation of Cells

The observation solution containing *T. pyriformis* was placed in a 35 mm glass-bottom dish (12 mm well, IWAKI, Chiyoda, Japan). The well with a volume of 12 mm in diameter and 1 mm in depth was filled with the suspension containing *T. pyriformis* for microscopic observation. The sample was set on an inverted microscope (Eclipse Ti; Nikon, Minato, Japan) equipped with a 2× objective (CFI Plan Apo λ, N.A. 0.10; Nikon, Japan) and an sCMOS camera (ORCA-Flash4.0; Hamamatsu, Japan) operated by NIS-Elements (Nikon, Japan). Bright field images were captured from the bottom interface to the top surface of the suspension, with an interval length of 50 μm. A set of about 20 shots was obtained within 15 s and repeated every 30 s for 10 min. Under this optical condition, the focal depth was calculated as approximately 90 μm from the size of the field of view of the camera, 6.75 mm per side with an image size of 2048 × 2048 pixels in 16 bit grayscale. As the cell was approximately 50 μm thick, the detectable depth of the cell was approximately 140 μm, which corresponded to 2–3 slices with 50 μm intervals. In the actual observations of the cells on the bottom glass, three slices detected them as in-focus cells. Because of this effective depth of detection and the curvature of the image plane resulting from the optics, the distribution of the detected cell population as a function of height from the bottom to the top of the sample was systematically broadened. The effects on cell counting caused by the observation setup mentioned above are discussed in the following section.

### 2.3. Counting of Cells

**Figure 1:**
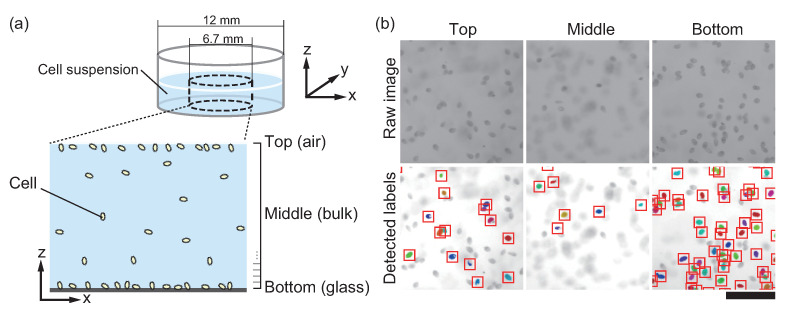
(**a**) Schematics of the experiment. *T. pyriformis* in a pancake-shaped region was observed. (**b**) Typical images captured by the microscope at different heights (top). The distribution of cells in the solution was captured every 50 μm from the bottom surface. The machine learning tool systematically recognized and counted only the cells on the focal plane (bottom). The scale bar is 400 μm.

Typical microscopy images of the suspension are shown in Figure 1. It is well-known that simple image processing, such as binarization, requires the careful tuning of a threshold to exclude out-of-focus cells. At times, the tuning encounters difficulties in identifying individual cells in nonuniform fields because luminance or background inhomogeneity frequently occurs in a crowded suspension. Although an adaptive threshold or further image processing can overcome these problems, image manipulations will arbitrarily change the detectable depth of focus. To avoid these problems, we used the automated area segmentation software, Trainable Weka Segmentation (TWS) [38], to identify in-focus cells. TWS learns certain training data cropped manually from an image and segments it automatically into two or more layers. We trained the classifier of the software to count only the cells on the plane of interest (Figure 1b), where several in-focus cell images were set for the training data; the same classifier was subsequently applied to all the images. The cells detected by TWS were subjected to image processing, and statistics of the number of individual cells were obtained through Analyze Particles of Fiji (https://fiji.sc/, accessed on 28 October 2021). Although systematic cell counting was achieved by the process mentioned above, the double or triple counting problem remained. We manually checked the number of detections per cell using several sets of images. For stationary cells at the interfaces, multiple counting factor 3 was the mode value. For moving cells in bulk, cases with factors 1 up to 6 or more emerged, but those with factors 2 up to 4 frequently appeared. Therefore, we adopted factor 3 to estimate the total cell number through all the cell concentrations. Note that the estimated factor affects only the total cell number, that is, the cell concentration (cells/mL) of each sample, and the number density of the cells. The concentration of cells (cells/mL) displayed in this paper was calculated as (raw counting number)/3 in the volume of 6.75 × 6.75 × 1.0 × π/4 mm^3^, considering the effective image circle where the corners of the square sensor were out of the field of view. In contrast, “the number of cells at the height (cells)” and “probability density of cell at the height”, the values of which are normalized such that they all sum to 1, were not affected by the multiple counting factor. The detected number of cells in a slice does not require division by 3; however, we should note that the effective detectable thickness of a slice was approximately 150 μm, the interval length of 50 μm times factor 3.

## 3. Results

### 3.1. *T. pyriformis* Distribute Close to Interfaces Rather than in Bulk Water

**Figure 2:**
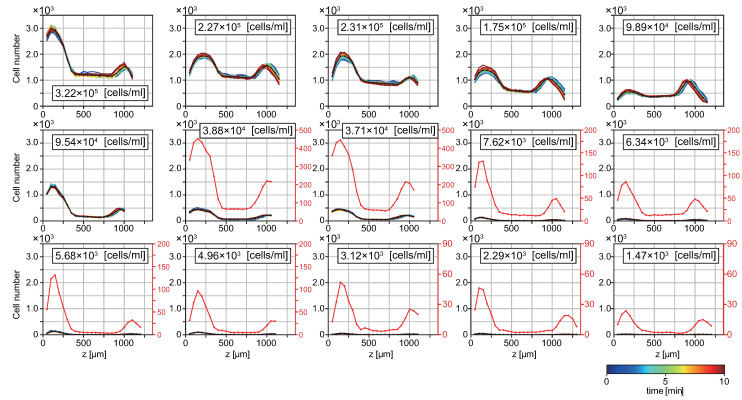
Spatial cell distribution of *T. pyriformis* suspension along the *z*-axis. Each plot displays the detected cell number as a function of height *z* from the bottom (*z* = 0). The cell concentrations of the suspensions are described in the respective panels. The color map indicates the time evolution (every 30 s for 10 min), and the black line represents the time average. To show the details of the lines that are squeezed near zero, we have added the red lines to the panels corresponding to the concentration equal to or smaller than 3.88 × 10^4^ cells/mL. The red line represents the same data as the black line with an expanded scale, as indicated by the red axis on the right side.

We obtained the spatial distributions of *T. pyriformis* by microscopy observation. Figure 2 and Figure S1 represent the number of cells along the *z*-axis, *N*(*z*). The height *z* = 0 corresponds to the solid surface of the glass-bottom dish. The value of cell density in each panel was estimated by dividing the sum of the cell number from the bottom to the top by the volume in the field of view, and then divided by the number of overlapping counts (factor 3, as mentioned above). The distribution of cell number *N*(*z*) has two sharp peaks near both air–liquid and solid–liquid interfaces, whereas it is almost flat and small in bulk. Note that the shape of the distribution near the water surface shifts slightly to the left with time because of the evaporation of the solution. Regardless of the cell concentration in the suspension, these distributions did not qualitatively change for 10 min, even though the cells were swimming continuously in the bulk water at 300 μm/s [17] and coming in and going out of the imaging area, exhibiting fluctuations of the value of the population. Because of small deviations among the distributions in each panel, it can be said that a steady distribution was realized. The accumulation of ciliates close to the interfaces was consistent with previous studies [17,39].

### 3.2. Probability of Accumulation Depends on Cell Density of Suspension

**Figure 3:**
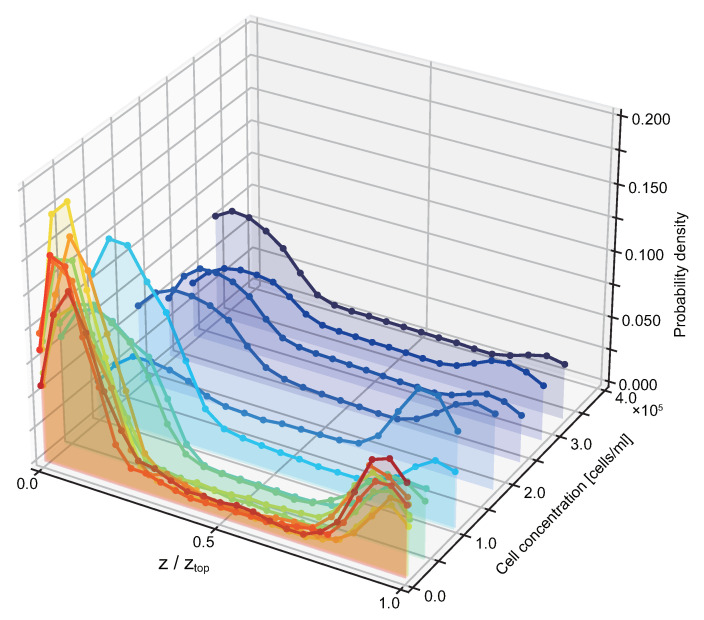
Probability density distribution of the cells as a function of relative z-position, *z*/*z*_top_, depending on cell concentration. For each plot line, the height axis is normalized with the height of the water from the bottom to the top surface, *z*/*z*_top_.

To compare cell distributions within suspensions with different cell concentrations, we normalized the cell number to calculate the cell probability along the *z*-axis (Figure 3 and Figure S2). For each concentration, the height *z* was normalized with the height of the water surface. As the cell concentration increased, the cell probability close to the interfaces decreased, and the difference in cell probability between the top and the bottom decreased as well. When cell concentration was lower than 0.5 × 10^5^ cells/mL, cell probability was approximately 0.15 and 0.075 at the bottom and top, respectively. In contrast, when cell concentration was higher than 1.0 × 10^6^ cells/mL, cell probability was approximately 0.060 and 0.050 at the bottom and top, respectively.

## 4. Discussion

### 4.1. Accumulation Coefficients

To quantify cell accumulation in the experiment, we defined two coefficients, α_solid_ and α_air_, as the degrees of accumulation at the bottom and top, respectively. α_solid_ is the ratio of the number of cells in the bulk to that at the bottom, and α_air_ is the ratio of the number of cells in the bulk to that at the top:

(1) $$\alpha_{\mathrm{solid}}=N_{\mathrm{solid}}/N_{\mathrm{bulk}},\;\;\alpha_{\mathrm{air}}=N_{\mathrm{air}}/N_{\mathrm{bulk}}.$$

**Figure 4:**
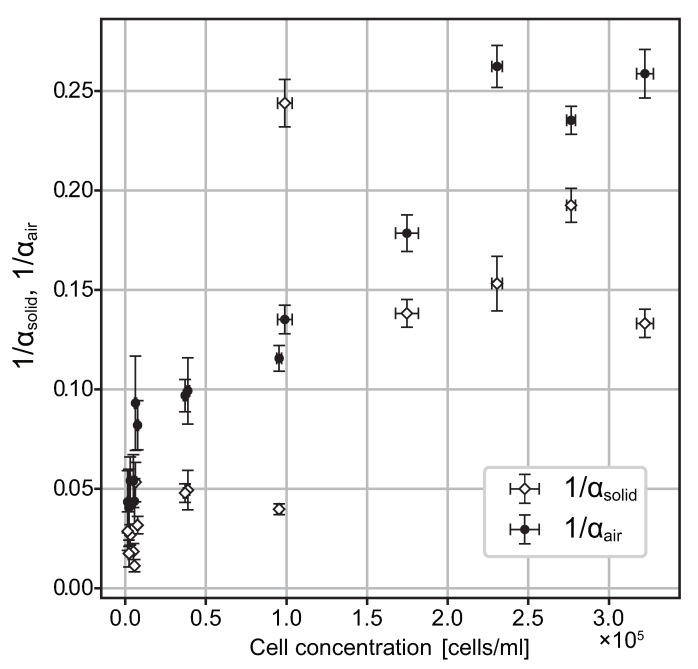
Accumulation coefficients calculated from experimental data. The error bars for the accumulation coefficients and cell concentration are due to the time average and dispersion of the 21 snapshots of the respective sample. The larger the coefficient, the larger the cell number ratio between the interface and the bulk.

In the present study, the accumulation coefficients at each cell concentration were calculated using the local spatially averaged cell number at the bulk, obtained from the experimental data (Figure 4). In the calculation, *N*_bulk_ was divided by factor 3 because of the experimental setup described in Section 2.2 and Section 2.3. In contrast, *N*_solid_ and *N*_air_ were adopted at the peak values of the solid–liquid and air–liquid interfaces, respectively. In consideration of Gaussian and delta distributions, the treatment mentioned above is one of the most reliable methods to describe the accumulation degree at and around the region. Note that it would rather overestimate the cell number in cases of broad peaks, as shown in the suspensions with higher concentration. Similar to the observations in Section 3.2, it can be seen from Figure 4 that as the cell concentration decreases, a larger percentage of cells are attracted near the solid–liquid and air–liquid interfaces. In most cases, α_solid_ was systematically larger than α_air_, which means that *T. pyriformis* prefers the solid–liquid interface to the air–liquid interface.

### 4.2. Diffusion Model

Spatio-temporal cell distribution can be described as a diffusion phenomenon that obeys Fick’s law [40]. When in bulk, cells move with equal probability in either the positive or negative direction of the *z*-axis. We assume the speed of the exodus from the solid–liquid interface to be a coefficient *m*_solid_, where *m*_solid_ = 1 is equal to the speed of the bulk, and those with >1 and <1 correspond to acceleration and deceleration, respectively. The same coefficient at the air–liquid interface is defined as *m*_air_. Therefore, a 1D diffusion equation for the cell population dynamics can be expressed as follows:

(2) $$\frac{dc_{j}\left(t\right)}{dt}=\left\{\begin{array}{cc} D\frac{m_{j+1}c_{j+1}\left(t\right)-m_{j}c_{j}\left(t\right)}{{\left(\Delta z\right)}^{2}}\; & \left(j=1\right)\\ D\frac{m_{j+1}c_{j+1}\left(t\right)-2m_{j}c_{j}\left(t\right)+m_{j-1}c_{j-1}\left(t\right)}{{\left(\Delta z\right)}^{2}}\; & \left(2\leq j\leq J-1\right)\\ D\frac{m_{j-1}c_{j-1}\left(t\right)-m_{j}c_{j}\left(t\right)}{{\left(\Delta z\right)}^{2}}\; & \left(j=J\right) \end{array}\right.$$

where *c*_*j*_(*t*) is the cell number or cell concentration at the spatial position *j*(*j* = 1, 2, ⋯, *J*) at time *t*, and *D* is the diffusion coefficient. The coefficients *m*_2_, ⋯, _*J* − 1_ = *m*_bulk_ = 1, and *m*_1_ and *m*_*J*_ correspond to *m*_solid_ and *m*_air_, respectively.

**Figure 5:**
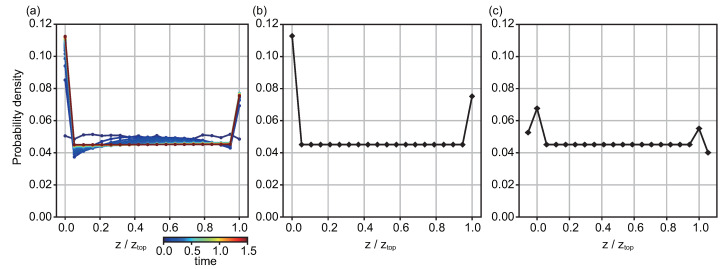
(**a**) Time evolution of the diffusion equation model by numerical simulation, where *D* = 0.001, Δ_*z*_ = 0.05, Δ_*t*_ = 0.005, and *T* = 300. The simulation starts with a random initial distribution and asymptotes to a bimodal stationary solution. (**b**) Analytical solution corresponding to the steady state of the system ${\vec{c}}_{\mathrm{steady}}$. (**c**) An example of the apparent distribution of cells considering the experimental setup generated by convoluting the analytical solution shown in (**b**).

If *m*_solid_ = *m*_air_ = 1, the cells have an equal probability of staying at the interface or moving to another position, and the resulting distribution of cells is flat. The coefficients with >1 and <1 indicate the depletion and accumulation of cells at the interface, respectively. For example, results similar to those shown in one of the experiments are obtained by numerical simulation of the equation and shown in Figure 5a. It is possible to represent a distribution with arbitrary degrees of accumulation and depletion at the interfaces.

This equation can be solved analytically using linear algebra (see Appendix A for details):

(3) $${\vec{c}}_{\mathrm{steady}}\propto \left[1/m_{\mathrm{solid}},\;1,\;\cdots ,\;1,\;1/m_{\mathrm{air}}\right]$$

The resulting steady solution coincides with the stationary distribution of the experiments or with that composed of the accumulation coefficients $\alpha_{\mathrm{solid}}$ and $\alpha_{\mathrm{air}}$ obtained from experiments without normalization constants:

(4) $$\left[1/m_{\mathrm{solid}},\;1,\;\cdots ,\;1,\;1/m_{\mathrm{air}}\right]=\left[\alpha_{\mathrm{solid}},\;1,\;\cdots ,\;1,\;\alpha_{\mathrm{air}}\right]=\frac{\left[N_{\mathrm{solid}},\;N_{\mathrm{bulk}},\;\cdots ,\;N_{\mathrm{bulk}},\;N_{\mathrm{air}}\right]}{N_{\mathrm{bulk}}}.$$

This model is useful for fitting the probability density of the experiment with the use of the relations α_solid_ = 1/*m*_solid_ and α_air_ = 1/*m*_air_. The analytically solved stationary distribution corresponds to the numerical result (Figure 5b)

In this steady-state solution, the cell number ratio between the bottom surface and bulk is indeed α_solid_, and the same is true for the top surface and bulk. Thus, the way of introducing *m*_solid_ = 1/α_solid_ and *m*_air_ = 1/α_air_ into the diffusion Equation (2) is consistent with the definition (1). More importantly, the accumulation coefficients introduced in this study can characterize the distribution of cells and have implications related to the microscopic population dynamics obeying the diffusion process.

### 4.3. Additional Discussion for the Model for Representing Experiment

The flux of cell migration can be measured and the accumulation coefficient from a microscopic point of view can be estimated by devising experimental methods. A diffusion coefficient not estimated from a stationary distribution of the cells can be estimated from the relaxation time from the initial homogeneous distribution of the cells to the steady distribution. In contrast, the flux and swimming speed of the swimmer also provide a diffusion coefficient through Fick’s law. Since the present experiments almost reached the respective steady distributions before the first image capture, and because the motion dynamics of the cells were not measured, the diffusion coefficient was not yielded experimentally.

To represent the actual distribution of cells, an experimentally yielded accumulation coefficient and a calculated distribution should be modified depending on the experimental setup. In the present case, the detectable thickness of a single shot was approximately 150 μm, which included three layers. In other words, the number of cells yielded in the experiment was a summation of those of the nearest neighbor layers. This situation was considered in the estimation of the accumulation coefficient, as in Section 4.1. Thus, the resulting distributions shown in Figure 5a,b are genuine distributions proposed numerically and mathematically. However, the apparent distribution yielded in the experiment strongly depends on the experimental setup. Under the present conditions, the apparent distribution of cells representing the results in Figure 2 requires the convolution of adding the number of cells in the nearest neighbor layers as shown in Figure 5c.

### 4.4. Comparison with the Suspension of Squirmer Model

As mentioned in the introduction, when a single neutral swimmer approaches the air–liquid or solid–liquid surface, it swims away from the surface due to hydrodynamic interaction. In contrast, a dense suspension of neutral swimmers (volume fractions of 10% and 40%, which correspond to extremely dense conditions in an actual experiment) packed between the surfaces tends to accumulate close to the surfaces [27]. Additionally, at high cell concentrations, the probability of neutral swimmers gathering close to the surfaces is lower than that at dilute cell concentration. Li and Ardekani mentioned that if many cells accumulate close to the surfaces, the cell number becomes saturated, and the probability in bulk relatively increases. They also indicated that crowded neutral swimmers increase their density near the wall, where the near-wall swimmers directed to the wall at nearly right angles. Since the cell densities of the present experiments (10^−5^–10^−3^ in volume fraction) were greatly lower than that of the previous simulation (0.1–0.4), there is little evidence to regard it as the mechanism of the present results, especially under the lower density conditions. It is a potent mechanism for the conditions of lower cell density that the ciliates exhibit sliding and staying on the wall at the single-cell level. Further research is needed to elucidate their exact contributions through measuring the swimming direction or body direction of all cells near the wall.

### 4.5. Biological Aspects

This study suggested that *Tetrahymena* aggregated at the air–liquid and solid–liquid interfaces under laboratory conditions, with a greater preference to solid–liquid interfaces. In nature, *Tetrahymena* congregates at solid–liquid interfaces such as the bottom of a lake [41]. As the bottom of a lake is rich in organisms and organic matter that *Tetrahymena* feeds on, we may attribute efficient predation on the surface to this behavior. In contrast, accumulation at the air–liquid interface is not often observed in nature [41]. Because the present experiment covered higher cell concentration than that in nature, it is therefore possible that the accumulation at the air–liquid interface was crucial in avoiding a lack of oxygen. Our study confirms that the unicellular organisms exhibited reasonable response for survival under conditions with insufficient oxygen.

## 5. Conclusions

We quantitatively measured the 3D spatial distribution of *T. pyriformis* in an open chamber. The cells accumulated both on the bottom solid–liquid interface and underneath the top air–liquid surface. These accumulations are large-scale verifications of the results of previous studies on solid–liquid [17,39] and air–liquid interfaces [15,16] and the quantification of ciliates in nature [41,42,43]. The intensity of accumulation was quantitatively defined as an accumulation coefficient. The coefficient can be estimated from a formulation based on a simple model and a simulation that can easily accommodate complex situations. While the accumulation coefficients at both interfaces were obviously higher than those in bulk under dilute suspension conditions, the relative value between those at the interfaces and those at the bulk became closer under dense suspension conditions. This systematic behavior is not negligible in the dynamics of highly concentrated microbial suspensions such as bioconvection, therefore it would be fundamental for designing efficient cell cultivation and applications in industrial technology and ecology. The accumulation coefficient suggested by the present study will assist future comparisons of results of *T. pyriformis* with those of different species. 

## Data Availability

All data needed to evaluate the conclusions in the paper are present in the paper and/or the Supplementary Materials.

## Supplementary Materials

The following are available online at https://www.mdpi.com/article/10.3390/mi12111339/s1; Figure S1: Logarithm plots of spatial cell distribution of *T. pyriformis* suspension as the function of height axis z with their time developments denoted by colors overwritten by their average in black lines; Figure S2: Probability density of number of cells as the function of the normalized height *z*/*z*_top_ depending on concentration of cells.

## Appendix A

Equation (2) can be rewritten using vector expression as follows:

(A1) $$\frac{d\vec{c}\left(t\right)}{dt}=\frac{D}{{\left(\Delta z\right)}^{2}}M\;\vec{c}\left(t\right)$$

where matrix *M* is given by

(A2) $$M\left(m_{\mathrm{solid}},m_{\mathrm{air}}\right)=\left(\begin{array}{ccccccc} -m_{\mathrm{solid}} & 1 & \; & & \; & & \\ m_{\mathrm{solid}} & -2 & 1 & \; & & \; & \\ \; & 1 & -2 & 1 & \; & & \\ \; & \; & \ddots & \ddots & \ddots & \; & \\ \; & \; & & 1 & -2 & 1 & \\ \; & \; & & \; & 1 & -2 & m_{\mathrm{air}}\\ \; & \; & & \; & & 1 & -m_{\mathrm{air}} \end{array}\right).$$

We can solve Equation (A1) immediately and obtain the following equation:

(A3) $$\vec{c}\left(t\right)=\exp\left[\frac{D}{{\left(\Delta z\right)}^{2}}Mt\right]\vec{c}\left(0\right)$$

Let *A* = *I* + *M*, then *A* is a column probability matrix (i.e., the sum of each column is 1) and has the following properties: (i) matrix *A* has an eigenvalue of 1; (ii) let the eigenvalue of *A* be λ, then |λ|≤1. Therefore, *M* has the following property: (i’) *M* has an eigenvalue of 0; (ii’) let the eigenvalue of *M* be κ, then κ≤0.

We therefore diagonalize matrix *M* as follows:

(A4) $$M=P\left(\begin{array}{cccc} 0 & \; & & \\ \; & \kappa_{2} & \; & \\ \; & \; & \ddots & \\ \; & \; & & \kappa_{J} \end{array}\right)P^{-1}$$

where 0 and $\kappa_{2},\cdots ,\kappa_{J}$ are the eigenvalues of matrix *M*, and the exponential factor in Equation (A3) is as follows:

(A5) $$\exp\left[\frac{D}{{\left(\Delta z\right)}^{2}}Mt\right]=P\left(\begin{array}{cccc} 1 & \; & & \\ \; & e^{D\kappa_{2}t/{\left(\Delta z\right)}^{2}} & \; & \\ \; & \; & \ddots & \\ \; & \; & & e^{D\kappa_{J}t/{\left(\Delta z\right)}^{2}} \end{array}\right)P^{-1}.$$

Since $\kappa_{2},\cdots ,\kappa_{J}<0$, the diagonal elements other than 1 will decay for a large *t*:

(A6) $$\exp\left[\frac{D}{{\left(\Delta z\right)}^{2}}Mt\right]\approx P\left(\begin{array}{cccc} 1 & \; & & \\ \; & 0 & \; & \\ \; & \; & \ddots & \\ \; & \; & & 0 \end{array}\right)P^{-1}.$$

Accordingly, the steady-state solution ${\vec{c}}_{\mathrm{steady}}$ is obtained as an eigenvector of matrix *M* corresponding to an eigenvalue of 0:

(A7) $$M{\vec{c}}_{\mathrm{steady}}=\vec{0}$$

which finally gives us Equation (3).
