# Supplementary material for: Accumulation of Tetrahymena pyriformis on Interfaces
Source: Micromachines (Basel). 2021 Oct 30;12(11):1339. doi: 10.3390/mi12111339 (PMC8622496; doi:10.3390/mi12111339)
Supplement: Supplementary file 1 [file micromachines-12-01339-s001.zip › micromachines-1419092-supplementary.pdf]

Supplementary Materials: Accumulation and Crowding Effect of *Tetrahymena pyriformis* on Interfaces

Kohei Okuyama [1], Yukinori Nishigami [2], Takuya Ohmura [3] and Masatoshi Ichikawa [1]*

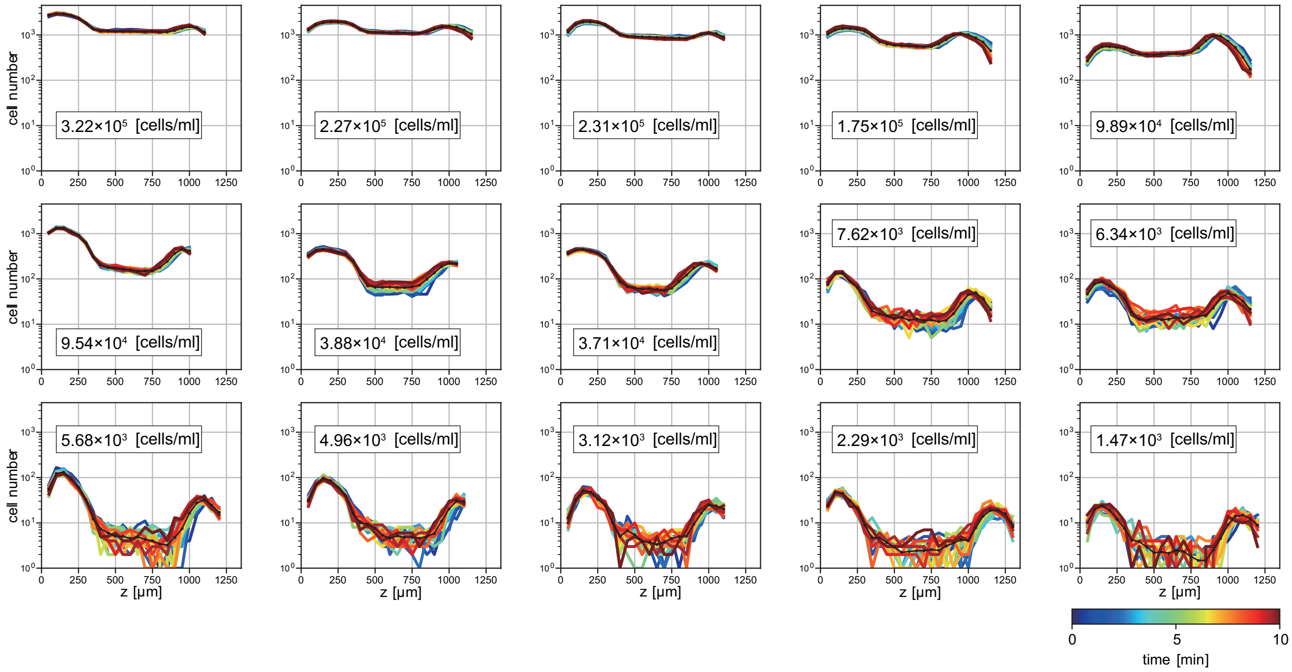

Figure S1. Logarithm plots of spatial cell distribution of *T. pyriformis* suspension as the function of height-axis z with their time developments denoted by colors overwritten by their average in black lines.

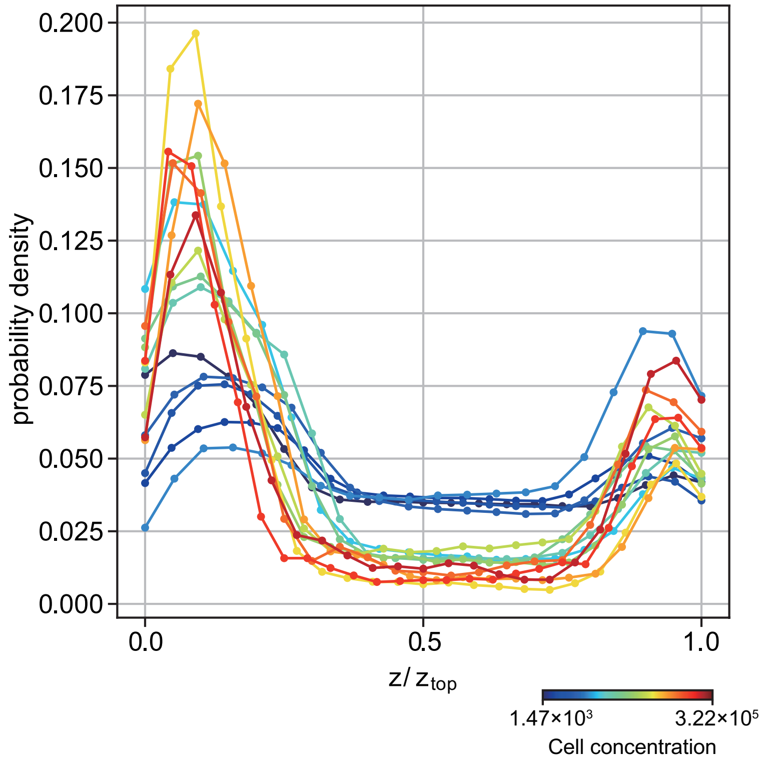

Figure S2. Probability density of number of cells as the function of the normalized height z/z_{top} depending on concentration of cells.
